# Family planning decision-making in relation to psychiatric disorders in women: a qualitative focus group study

**DOI:** 10.1186/s12978-024-01836-8

**Published:** 2024-07-02

**Authors:** Shahenda A. I. H. Ahmad, Jorina Holtrop, Monique J. M. van den Eijnden, Nini H. Jonkman, Maria G. van Pampus, Odile A. van den Heuvel, Birit F. P. Broekman, Noralie N. Schonewille

**Affiliations:** 1 Department of Psychiatry and Medical Psychology, OLVG, Oosterpark 9, Amsterdam, 1091 AC The Netherlands; 2 Team Knowledge, Innovation and Research, MIND, Stationsplein 125, Amersfoort, 3818 LE The Netherlands; 3 Department of Research and Epidemiology, OLVG, Oosterpark 9, Amsterdam, 1091 AC The Netherlands; 4 Department of Gynecology and Obstetrics, OLVG, Oosterpark 9, Amsterdam, 1091 AC The Netherlands; 5 Department of Psychiatry, Amsterdam UMC, Vrije Universiteit Amsterdam, Boelelaan 1117, Amsterdam, 1081 HV The Netherlands; 6 Department of Anatomy and Neuroscience, Amsterdam UMC, Vrije Universiteit Amsterdam, Boelelaan 1117, Amsterdam, 1081 HV The Netherlands; 7 Compulsivity, Impulsivity and Attention Program, Amsterdam Neuroscience, Amsterdam, The Netherlands; 8 Amsterdam Public Health, Mental Health Program, Amsterdam UMC, Vrije Universiteit Amsterdam, Boelelaan 1117, Amsterdam, 1081 HV The Netherlands

**Keywords:** Family planning, Unintended pregnancies, Psychiatric disorders, Decision-making, Qualitative research

## Abstract

**Background:**

Recent studies revealed an elevated likelihood of unintended pregnancies among women with psychiatric disorders compared to their counterparts without such vulnerability. Despite the importance of understanding family planning decision-making in this group, qualitative inquiries are lacking. This study explored family planning decisions among women with psychiatric disorders.

**Methods:**

Utilizing a qualitative approach, three focus group discussions were conducted with purposive sampling: women with a history of unintended pregnancies (*N* = 3), women without children (*N* = 5), and women with a history of intended pregnancies (*N* = 9), all of whom had self-reported psychiatric disorders. Using thematic framework analysis, we investigated the themes “Shadow of the past,” reflecting past experiences, and “Shadow of the future,” reflecting future imaginaries, building upon the existing “Narrative Framework.”

**Results:**

The Narrative Framework formed the foundation for understanding family planning among women with psychiatric disorders. The retrospective dimension of focus group discussions provided opportunities for reflective narratives on sensitive topics, revealing emotions of regret, grief and relief. Childhood trauma, adverse events, and inadequate parenting enriched the "Shadow of the past". The “Shadow of the present” was identified as a novel theme, addressing awareness of psychiatric disorders and emotions toward psychiatric stability. Social influences, stigma, and concerns about transmitting psychiatric disorders shaped future imaginaries in the shadow of the future.

**Conclusions:**

This study enlightens how family planning decision-making in women with psychiatric disorders might be complex, as marked by the enduring impact of past experiences and societal influences in this sample. These nuanced insights underscore the necessity for tailored support for women with psychiatric disorders.

**Supplementary Information:**

The online version contains supplementary material available at 10.1186/s12978-024-01836-8.

## Abstract (plain language summary)

Recent studies show that women with psychiatric disorders are more likely to experience unintended pregnancies. However, the underlying reasons are not fully understood. Understanding those reasons is important to provide better healthcare. Our study explored how women with psychiatric disorders make decisions about family planning.

We had conversations with different groups of women—women with unintended pregnancies, women without children, and women with intended pregnancies—through focus group discussions. We partnered with the Dutch mental health organization MIND to capture diverse opinions. Key themes and categories in the discussions were identified and organized.

We found four main themes: "Shadow of the past" showed how past events, trauma, and lack of knowledge about parenting affect family planning. "Shadow of the present" revealed different feelings about family planning, the importance of the awareness of psychiatric disorders, and uncertainty about decisions. "Shadow of the future" included thoughts about becoming a mother, the impact of social influences, and concerns about passing on psychiatric disorders. "Reflections on the decision" showed how psychiatric disorders, experiences with motherhood, and feelings of regret, grief and relief had an influence on family planning decisions.

In conclusion, our study highlighted the complexity of family planning decisions for women with psychiatric disorders. Past experiences and societal influences, like stigma, play a big role. These insights show the need for personalized family planning support for women with psychiatric disorders.

**Supplementary Information:**

The online version contains supplementary material available at 10.1186/s12978-024-01836-8.

## Background

Literature suggests that psychiatric disorders and family planning decision-making are related. Recent studies revealed that women with psychiatric disorders more often experience unintended pregnancies compared to counterparts without such vulnerability [1–3]. Moreover, childlessness is associated with having chronic illnesses, among which are psychiatric illnesses [4, 5]. However, there is limited knowledge about contributing factors that shape family planning decision-making in women with psychiatric disorders [6, 7].

Family planning decision-making defines the process through which individuals make choices about whether to have children, when to have them, and how many children to have [8]. It involves contemplating economic, social, cultural, and health-related factors [9]. Understanding family planning decision-making is fundamental for estimating the need for contraception, predicting reproductive patterns, and developing programs aimed at preventing unintended pregnancies [10]. The use of (emergency) contraceptives [11], improved accessibility to abortion services [12], and increased understanding of risk factors [11, 13, 14] have reduced unintended pregnancies. However, in 2010–2019, more than half of all pregnancies worldwide were still unintended [15].

Unintended pregnancies are particularly common among women with psychiatric disorders, reaching rates of up to 65% [5, 16]. Aside from difficulties with planning, women with psychiatric disorders face elevated risks of psychiatric problems after pregnancy, leading to additional risks for adverse outcomes for both mothers and children [17–20]. The preconception phase is crucial for women planning to conceive by offering an opportunity to enhance nutrition and lifestyle choices to minimize maternal and child health risks [21]. Pregnancy planning is especially important for women with psychiatric disorders because they may need to make medication adjustments, take precautionary measures regarding the relapse of psychiatric disorders, and optimize mother–child attachment [17–20]. Indeed, women with unintended pregnancies encounter significant additional challenges due to the absence of the pregnancy planning phase, such as limited access to prenatal care, financial strain, and emotional stress [1].

Several frameworks exist for describing the factors that shape family planning decision-making [22–28]. However, most frameworks are past-driven and focus predominantly on cognitive factors [29]. We hypothesize that these frameworks are inadequate for capturing the uncertainty about the future faced by women with psychiatric disorders. The “Narrative Framework” provides a different perspective on family planning decision-making, particularly amid the amplified uncertainty and stress of the COVID-19 pandemic. It integrates past experiences, psychological predispositions, and socioeconomic factors, termed the “Shadow of the past,” alongside expectations, future imaginaries, and future narratives, termed the “Shadow of the future.” This framework captures decision-making processes by including these elements.

The aim of this qualitative study was to delve into the family planning experiences of women with psychiatric disorders (history of psychiatric disorder and/or current diagnosis). The “Narrative Framework” will provide a foundation for structuring the themes involved in the decision-making process [29]. The results of this study will contribute to knowledge about family planning decision-making in women with psychiatric disorders.

## Materials and methods

### Study design

A qualitative study of experiences with family planning in women with self-reported psychiatric disorder(s) was conducted. We adopted a constructionist approach in which we acknowledged the role of society in shaping perspectives on family planning decision-making [30]. Therefore, focus group discussions were selected as the method of data collection because participants themselves can represent societal influences on each other’s perspectives.

### Participant recruitment

**Fig. 1:**
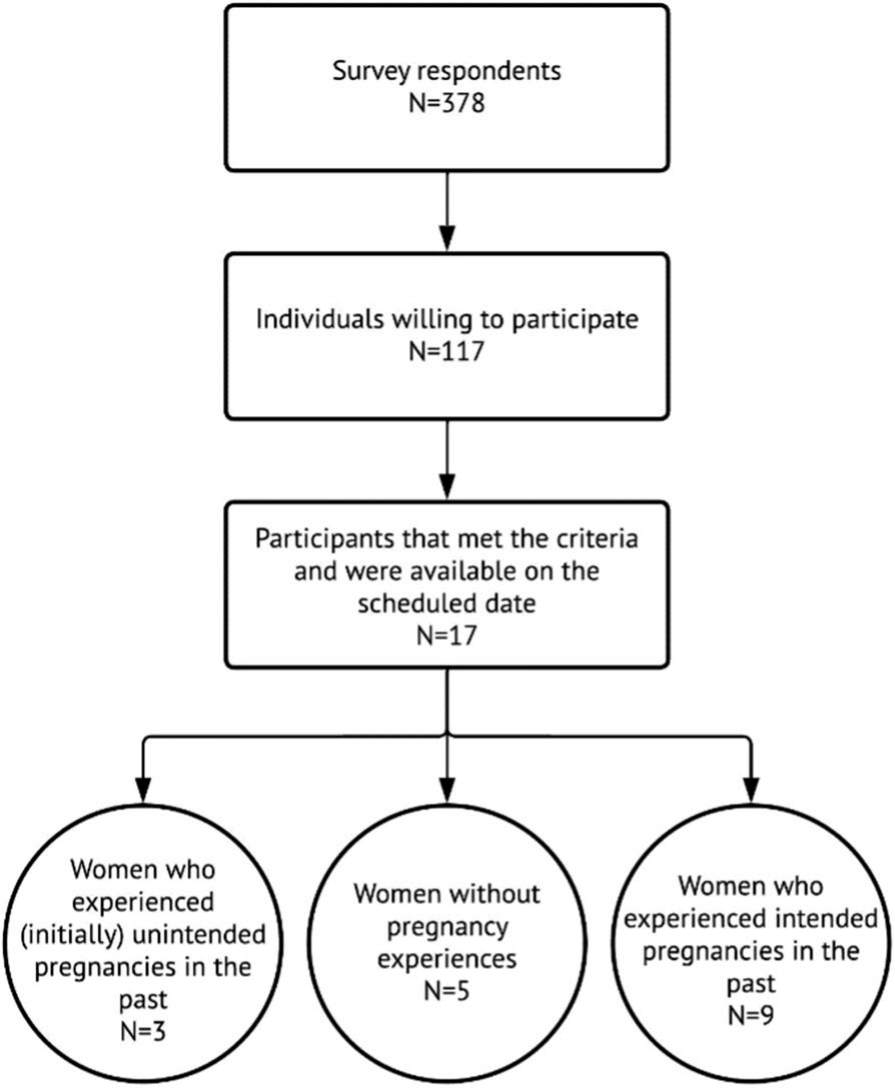
Flowchart of participant inclusion. Legend: this figure outlines the participant selection process for the study

Seventeen volunteers of the Dutch mental health umbrella organization MIND with self-reported psychiatric disorders were included. Participants were recruited from a sample of survey respondents (*n* = 378) from MIND [5]. The survey was conducted to collect quantitative and qualitative information about family planning. Participants were given the option to provide their email address if they wished to participate in a focus group discussion. Both men and women participated in the survey, but to address the current research question, only women were eligible (*n* = 17). Prior to participating in the focus groups, and after explanation of the study, all participants signed an informed consent form. Focus group discussion 1 (*n* = 3) consisted of women with a history of (initially) unintended pregnancies. Women who experienced an unintended pregnancy but did not remain pregnant (due to miscarriage or abortion) also participated in focus group discussion 1. Focus group discussion 2 (*n* = 5) consisted of women who did not have children and who had not been pregnant prior to participation. Focus group discussion 3 (*n* = 9) consisted of women with a history of intended pregnancies resulting in one or more children. Purposeful sampling created homogeneous focus group discussions regarding pregnancy intentions, ultimately benefiting the willingness of women to engage in discussions [31]. Due to the anonymous nature of the survey, it was unclear prior to the focus group discussions which psychiatric disorders the participants had been diagnosed with. Medical records were inaccessible; participants disclosed their psychiatric diagnoses during the focus group discussions. Figure 1 provides an overview of participant inclusion.

### Data collection and storage

Three focus group discussions were held at a central location in the Netherlands (organization MIND, Amersfoort, The Netherlands) between October and November 2021. The interview guide was created based on answers to an earlier survey and consisted of two main research questions, complemented by specific questions per focus group discussion: 1) How does your (history with) psychiatric disorder influence your desire for children? and 2) What is your experience discussing family planning with your mental health professional? The interview guide for each focus group is provided in Additional file 1. The focus group discussions were held in Dutch and lasted between 120 and 155 min (median duration 123 min). A researcher with lived experience with perinatal mental health problems [ME] led the discussions, [NS] was present to observe and take field notes. Digital research data, including audio recordings and ad verbatim transcripts of the focus group discussions, were pseudonymized and stored in a password-protected file on a secure server of the hospital (OLVG). Paper consent forms are stored in a locked research cabinet of the same hospital. This process ensures that the data is securely stored, and that participants’ anonymity is protected throughout the study.

### Data analysis

The focus group discussions were audio-recorded and transcribed ad-verbatim. The transcripts were converted to ATLAS.ti v9 for data analysis. Table 1 summarizes the steps performed during the analysis.

**Table 1:** Thematic framework analysis process

↑ Inductive	Step 1	Self-acquainting with the data, by reading and rereading of the transcripts [JH, NS, SA, YD, ME]
	Step 2	Line-by-line analysis and inductive coding by three researchers independently [NS, SA, YD]. [NS] and [YD] performed the coding process directly after the focus group discussions, while [SA] performed the coding process later. Open codes with a low interpretation degree were applied
	Step 3	Merging of similar codes by one researcher [SA] by which the quantity of the codes was reduced
	Step 4	Excluding codes with no relation with the family planning decision-making process by two researchers [JH, SA]. A step known as selective coding
Deductive ↓	Step 5	The selective codes were applied to the “Narrative Framework” (29) by two researchers independently [SA, JH]
	Step 6	A within-person analysis was performed by charting the selective codes for every participant individually in the framework. This was done by two researchers separately [JH, SA]. Codes that were not applicable to the preexisting themes (shadow of the past and shadow of the future) were discussed during group meetings and later identified as new themes (shadow of the present and reflections)
	Step 7	A between-person analysis was carried out through axial coding by two researchers individually [JH, SA], producing categories. Categories were discussed in several group meetings resulting in a new framework visualizing four overarching themes and several categories

This table presents the thematic framework analysis, showing the inductive steps (1-4) and deductive steps (5-7) taken in the analysis process

### Methodological integrity

The research team, with backgrounds in psychiatry, obstetrics, neurosciences, and health behavior, ensured a foundation for conducting a nuanced and in-depth qualitative analysis of family planning decision-making, thereby incorporating triangulation. The epistemological approach was clearly stated and closely adhered to, which helped to align the research question with the applied methods. The conclusions were grounded in the evidence through the inclusion of quotations. Providing contextual information, such as the study setting and participant details, enhanced the comprehensibility of the results. Unlike a conventional consensus-reaching method, different interpretations were integrated into the findings to enrich the data analysis process. Utilizing methods of researcher reflexivity, such as memos and field notes, contributed to a reflexive and transparent analytical process. Reflexivity was considered throughout the process, acknowledging that [ME]’s background with lived experience with perinatal mental health problems may have influenced the discussions and interaction with participants, potentially fostering a more open and empathetic environment.

## Results

### Demography

Information about the demographics of the participants (*n* = 17) is reported in Table 2. Ages ranged between 24 and 70 years, with a median age of 57 years. All women had a Dutch background. The participants exhibited diverse occupational backgrounds, with six (35%) declaring themselves unfit for employment for reasons related to their psychiatric disorder(s). A history of pregnancy and psychiatric disorder(s) are described in Table 3. Mood disorders were the predominant psychiatric disorder (*n* = 10), manifesting across all focus group discussions. Subsequently, trauma-related disorders (*n* = 8) and anxiety disorders (*n* = 6) were the most prevalent.

**Table 2:** Demographic features of participants

Demographic characteristic	Category	Focus group discussion		
		Women with (initially) unintended pregnancies (*n* = 3)	Women without children (*n* = 5)	Women with intended motherhood (*n* = 9)
**Age**	20–30	0	2	0
	31–40	0	0	2
	41–50	0	1	1
	51–60	0	2	2
	61–70	2	0	3
	Unknown^a^	1	0	1
**Occupation**	Employed	0	2	2
	Unemployed	0	1	0
	Declared unfit for employment	0	2	4
	Unknown^a^	3	0	3

^a^Information may not be available for all participants, as noted by the ‘unknown’ lines

This table presents the demographic characteristics of participants (*n* = 17), including participants per age category and employment status in each focus group discussion

**Table 3:** History of pregnancy and psychiatric disorder(s) of participants

Demographic characteristic	Category	Focus group discussion		
		Women with (initially) unintended pregnancies (*n* = 3)	Women without children (*n* = 5)	Women with intended motherhood (*n* = 9)
**History of pregnancy**	Primipara	1	0	5
	Multipara	2	0	4
	Abortion or miscarriage	2	0	1
**Parent**	Yes	2	0	9
	No	1	5	0
**(History of) psychiatric disorder per category**^**a**^	Anxiety disorders	1	0	5
	Mood disorders	2	4	4
	Psychotic disorders	0	0	1
	Personality disorders	1	0	1
	Neurodevelopmental disorders	1	5	3
	Trauma related disorders	3	2	3

^a^Multiple psychiatric disorders per person are possible, all participants described ≥ 1 disorder

This table presents the demographic characteristics of participants (*n* = 17), including obstetric (pregnancies and parenthood) and psychiatric disorder history in each focus group discussion

### Framework

**Fig. 2:**
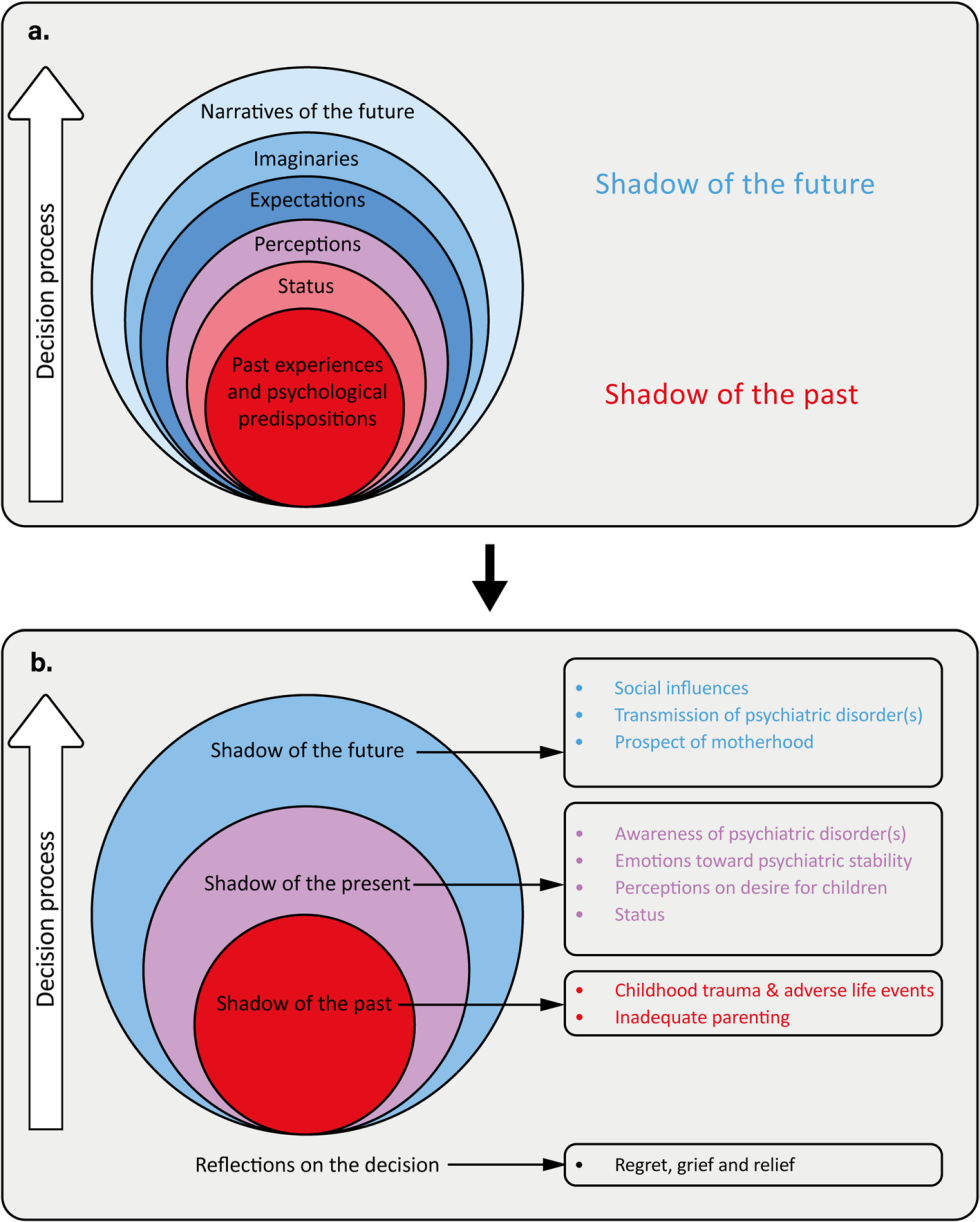
Framework of family planning decision-making in women with psychiatric disorders. Legend: this figure shows the framework of family planning decision-making with part (**a**) illustrating the Narrative Framework and part (**b**) illustrating the adapted version tailored to women with psychiatric disorders. For each theme categories are listed on the right side of figure b. This figure is reproduced with permission from “Guetto R, Bazzani G, Vignoli D (2022) Narratives of the future and fertility decision-making in uncertain times. An application to the COVID-19 pandemic. Vienna Yearb Popul Res 20:223–260. https://www.jstor.org/stable/27222579”

Our findings are presented within the context of an adapted version of the “Narrative Framework”. While the original framework highlights “Shadow of the past” and “Shadow of the future” as primary themes, our findings expanded this framework by incorporating two additional themes, namely “Reflections on the decision” and “Shadow of the present”, which were specifically tailored to our study population. This adaptation is illustrated in Fig. 2. Each theme includes categories supported by quotations translated into English. Additional file 2 provides the original Dutch quotations.

### Reflections on the decision

The retrospective nature of the focus group discussions allowed 17 women to delve into reflective narratives concerning sensitive topics regarding their psychiatric disorder(s) and family planning decision-making. The importance of these reflections, amplified by their emotionally charged nature, justifies the creation of a dedicated theme to them, as they were not yet addressed by the Narrative Framework [29]. Table 4 provides several quotations belonging to this theme.

**Table 4:** Quotations belonging to the theme reflections on the decision

Regret, grief and relief	***57 years, two daughters:*** “From the age of forty-eight, I became conscious of the fact that I have been psychiatrically vulnerable since my youth. It has cast a significant shadow over the pregnancies and childbirths.” ***61 years, one daughter:*** “Because in 2012 my daughter, who is now 30, suffered from severe depression and she has still not recovered from it. And if I had known that in advance, I also have depression, I am also in depression now. Then she would never have been born… If I had known that she would develop such a severe depression (other participant: that you passed on). Yeah, I probably passed that on. And I find that very terrible.” ***53 years, no children:*** “At a certain point I noticed that I had Tourette’s, and there are all kinds of complaints associated with it. And then I was very happy because of heredity that I didn’t do it [have a child]. It remains painful sometimes, it always remains a sore spot somewhere. Yes, very happy, and very sorry, it’s just a shame sometimes.” ***70 years, two sons, experienced an unintended pregnancy*:** “When I look back on my life, I am now seventy, those were my golden years [with the children]. It was very busy and I had to take a lot of care, having four hands at the same time, but I did it.” ***59 years, no children:*** “Give me the box of tissues [crying and laughing]. My psychiatrist always says to me, because we have talked about it [not having children], you have taken very good care of your children. You have kept them well.”

This table shows quotations belonging to the theme Reflections on the decision, grouped by the category: Regret, grief, and relief

#### Regret, grief and relief

Reflections on family planning decisions revealed a mix of emotions experienced by the participants. Participants without children often felt grief, yet some also found relief in their decision. This illustrates the complexity of emotions within individuals. The impact of participants' psychiatric disorder(s) on family planning was central in all focus group discussions. For some women, challenges in parenting due to personal circumstances brought feelings of grief and regret. However, positive reflections on motherhood also emerged, particularly from mothers who found fulfillment in motherhood despite initially unintended pregnancies.

### Shadow of the past

Originally, the shadow of the past reflected the impact of life experiences, psychological predispositions, and socioeconomic factors on an individual’s decision about having children [29]. In our sample, this theme was enriched by the interplay between personal experiences in the women’s upbringing, the perceived parenting skills of their parents, and how those women respond to these experiences. Quotations are provided in Table 5.

**Table 5:** Quotations belonging to the theme shadow of the past

Childhood trauma and adverse life events	***62 years, no children, experienced an unintended pregnancy:*** “Even though I never really felt at home in my parental home, I still wanted something of a family.” ***53 years, no children:*** “I come from a German family, which also caused war trauma. And then I think, what are you passing on, apart from the technical story of passing on. That is my justification [for not having a child].”
Inadequate parenting	***32 years, one son:*** “Yes, I had that too. That you did have an [bad] example. I was like, I don't want to do it like my parents … I want to do it differently.” ***59 years, no children:*** “My father had a mood disorder, manic depressive [disorder]. … My mother has ADD, or ADHD, so I see it from both sides. I see my mother running back and forth hysterically, and I know my father has had huge lows. And then I look back and I’m glad that I didn’t have any children.”

*ADD* attention deficit disorder, *ADHD* attention deficit hyperactivity disorder

This table shows quotations belonging to the theme shadow of the past, grouped by the categories: childhood trauma and adverse life events, and inadequate parenting

#### (Childhood) trauma and adverse life events

The participants of all focus group discussions spontaneously shared (childhood) trauma and adverse life events when asked about their family planning decision-making, revealing their perceived connection between these experiences and their family planning considerations. They believed that their upbringing impacted their decisions. Lived experience with trauma affected perspectives on family planning differently: some women were motivated to move past their childhood trauma by building a (better) family for themselves by having a child, while other women refrained from having children because of their adverse life events.

#### Inadequate parenting

This category showed how reflections on their own upbringing, specifically on their parents' parenting skills or the lack thereof, can influence women’s perspectives on having children. The decision to have children became a personal and reflective process shaped by the desire to replicate positive aspects of one's upbringing or breaking away from negative patterns and challenges.

### Shadow of the present

In the Narrative Framework [29], (socioeconomic) status and personal perceptions bridged the gap between the shadow of the past and the shadow of the future. While socioeconomic factors such as financial considerations or the availability of a (suitable) partner contributed to the decision-making process of the participants, personal perceptions of their psychiatric disorder(s) were pertinent. This led to the extension of the framework with a novel theme: the shadow of the present. This theme incorporates categories relevant to our sample, including awareness of psychiatric disorder(s) and psychiatric stability, which were previously unaddressed in empirical models of family planning (Table 6).

**Table 6:** Quotations belonging to the theme shadow of the present

Awareness of psychiatric disorder(s)	***70 years, two sons, experienced an unintended pregnancy:*** “I had children at a time when I wasn’t very aware of my psychiatric disorders. But I always knew that I was different.” ***47 years, two sons:*** “I had myself diagnosed [with autism], and that immediately explained a whole lot, why things were always so challenging, including motherhood, which was a bit more difficult for me than for most parents of my children's friends.”
Emotions toward psychiatric stability	***61 years, one daughter:*** “I developed a psychosis at the age of eighteen and yet I dared to get pregnant around the age of thirty. I thought, I can handle this.” ***40 years, no children:*** “I've always known no children for me because then the whole mess [depression] would repeat itself, I'm not going to do that.”
Perceptions on desire for children	***70 years, two sons, experienced an unintended pregnancy: “***The fact that [name son] was born, was in my case, a conscious choice. (Interviewer: but did you have doubts about the decision?). I had some doubts; I did not actually know what I wanted [laughing]. I did not even know what I wanted to do with my life.” ***62 years, no children, experienced an unintended pregnancy:*** “Maybe that's why I can't come to terms with it [unintended pregnancy] emotionally. It's a lot. Just traumatized.”
Status	***40 years, no children:*** “I have had a very stable partner for about four years now, and now I sometimes think a bit [of wanting a child], but I am forty, so…”

This table shows quotations belonging to the theme shadow of the present, grouped by the categories: awareness of psychiatric disorder(s), emotions toward psychiatric stability, perceptions on the desire for children, and status

#### Awareness of psychiatric disorder(s)

Awareness of psychiatric disorder(s) at the time of the decision was a recurrent theme among participants with children. Some participants mentioned the impact of not being aware of their psychiatric disorder at the time of the decision, indicating that they would have made different choices if they had been aware of the diagnosis earlier. For some of the participants, the diagnosis was liberating, explaining the challenges of motherhood.

#### Emotions toward psychiatric stability

Psychiatric stability at the time of decision-making was mentioned as one of the factors influencing choices. For some individuals, the stability of symptoms presented an opportunity to pursue parenthood, while for others, psychiatric stability did not hold the same level of deliberative weight. Diverse attitudes toward this issue highlight how some participants experienced resilience by learning from previous experiences, while others did not.

#### Perceptions of desire for children

A spectrum of diverse viewpoints on the desire for children was described as a complex array of thoughts, emotions, and perspectives that concurrently coexist, contributing to a fluctuating experience over time and giving rise to feelings of ambivalence and uncertainty. As one participant expressed “childbearing desire is not 100% yes or 100% no,” highlighting nuanced attitudes toward motherhood and childlessness. While ‘perceptions’ were originally emphasized as personal interpretations of past and current experiences [29] our participants argued that viewpoints on the desire for children change over time.

#### Status

Opportunities and constraints for childbearing plans resemble the (socioeconomic) status element of the Narrative Framework [29]. This category includes several factors mentioned as reasons whether to have a child, including maternal age, financial stability or having a (stable) partner.

### Shadow of the future

The shadow of the future emphasizes the importance of expectations and personal narratives in uncertain situations [29]. Our study shows how social influences, stigma around mental health, and uncertainty about passing on a condition can influence future imaginaries (Table 7).

**Table 7:** Quotations belonging to the theme shadow of the future, grouped by the categories: social influences, transmission of psychiatric disorder(s), and the prospect of motherhood

Social influences	***24 years, no children:*** “Yes, I don’t have that much support from the family. So, then I think, why [would I have child].” ***53 years, no children:*** “My best friend took over my favorite name for a daughter, that has happened twice now. “you're not having children anyway” I just think that’s so inconsiderate. And I'm glad that I only now know that I have autism, because people have a prejudice, like I couldn’t do that [be a mother]. While that doesn’t have much to do with it. I know plenty of people with autism who can take excellent care of their children.” ***age unknown, one son:*** “And what I found very difficult, was that people automatically assumed that I did not want to keep the child. I found that difficult. While for me that is not a question at all.”
Transmission of psychiatric disorder(s)	***29 years, no children:*** “I wouldn’t want to bring a child into the world who might inherit some of my psychiatric complaints.” **53 years, no children**: “It is a line that had to be stopped (Interviewer: Yes, the past, whether that continues, and whether you still want to create something new for yourself, right?) … Yes, I now have more self-confidence and knowledge about that, that it is possible (*other participant:* to break the cycle of intergenerational transmission).”
Prospect of motherhood	***59 years, no children:*** “I don’t think I could have raised them [children] well in the years before.” ***29 years, no children:*** “Can I be a good parent? I wonder if I could give a lot of love. … I don’t think that I could really be a good mother. So yes, also a bit out of protection, I think.” ***53 years, no children:*** “No one is 100% successful in raising children. There are also people without any history [of psychiatric disorder] where things go terribly wrong. And of course, you never know what will come your way.” ***32 years, one son:*** “Thanks to the knowledge I now have, I can say clearly that I have a heavy genetic burden. And that is of course also something that I am now more aware of, and “what if my child gets that” goes through my mind. On the other hand, I can say that I have it myself and I now know very well how to deal with it, so I probably recognize it sooner (*other participants:* yes, yes, yes) and I can also provide better support if so. That makes that I don’t doubt myself as a mother.”

This table shows quotations belonging to the theme shadow of the future, grouped by the categories: social influences, transmission of psychiatric disorder(s), and the prospect of motherhood

#### Social influences

During the focus group discussions, the social system’s impact was heavily discussed. Many expressed frustrations with the lack of support from their social environment for their desire for children, leading to uncertainty about their decision. While personal visions of the future can be influenced by society, personal visions may also differ, thereby placing social influences in the shadow of the future [29]. Participants’ experiences with stigma due to their psychiatric disorder(s) often leaned toward deciding against having children.

#### Transmission of psychiatric disorder(s)

The transgenerational transmission of psychiatric disorder(s) to children was a key theme among participants, as discussed in all focus group discussions. Many participants were conscious of the risk of passing their condition to their offspring, which influenced their decisions against having children or causing regret if transmission occurred. Awareness of the challenges varied, with some participants doubting the possibility of breaking the transmission cycle, while others remained hopeful.

#### Prospect of motherhood

Insecurities about motherhood were deliberated. Participants felt incapable of raising a child for varying reasons, such as difficulty combining motherhood and having psychiatric symptoms. Other participants could rationalize this by referring to ‘other mothers’ without psychiatric disorder(s) who make parenting mistakes. Another participant stated that although her illness was heritable, it also aided her in supporting her child.

## Discussion

### Key findings

This study has provided insights into family planning decision-making among women with psychiatric disorders by extending the Narrative Framework [29] with two themes. First, we dedicated a theme to reflections on decision-making, which encompasses emotions of grief, relief and regret. Second, we introduced the shadow of the present, which emphasized the impact of psychiatric disorders on decision-making by considering awareness of psychiatric disorders and psychiatric stability. Furthermore, the shadows of the past and future were broadened by integrating categories tailored to women with psychiatric disorders, including trauma, adverse life events, and social influences.

### Interpretation in relation to literature

The retrospective nature of the focus group discussions allowed us to reflect on the participants’ family planning decisions, where the emotions of regret, grief and relief emerged. Regret over the delay in childbearing decisions has been described before in couples seeking fertility treatments [32]. Like our participants, voluntary childless women reported relief, feeling financially unburdened compared to their parenting peers, and enjoying various forms of freedom. However, they also faced stigmatization, and some expressed that their decision was influenced by their concern about potentially transmitting diseases to their children [33]. We hypothesize that cognitive dissonance, influenced by the type of psychiatric disorder, may contribute to regret in individuals as they grapple with conflicting thoughts and emotions [34].

Our study enhanced the shadow of the past with insights from 17 women with psychiatric disorder(s), shedding light on the impact of (childhood) trauma and adverse experiences on family planning decision-making. Previous research has shown an increased risk of unintended pregnancy in mothers with adverse childhood experiences [35]. Furthermore, women with unintended pregnancies reported more psychosocial problems [36]. Together with our findings, these findings imply that past experiences (related to psychiatric disorders) play a significant role in shaping family planning decisions and outcomes.

In the shadow of the present, we expanded upon the existing themes of (socioeconomic) status and perceptions [29]. Various enablers and constraints in the decision-making process surfaced, aligning with descriptions in other frameworks [22, 23, 25, 26], and are therefore not unique to our population. The personal perceptions of our participants were portrayed as a complex array of emotions and thoughts, contributing to a fluctuating experience of family planning marked by ambivalence and uncertainty. Ambivalence toward motherhood in women with severe mental illness has been previously documented [37]. However, our study focused primarily on ambivalence in decision-making. Surprisingly, participants did not bring up the issue of psychoactive medication usage in relation to family planning. Although most psychotropic medication can be continued during pregnancy, some psychoactive medications can be teratogenic and should be used with caution [38, 39]. Also, previous studies showed that (pregnant) women with psychiatric disorders contemplate their medication usage [40]. Overall, our findings suggest that family planning decision-making is more complex in women with psychiatric disorders than in those without psychiatric disorders, consistent with prior research [41]. A potential explanation lies in additional factors influencing the decision, such as awareness of the psychiatric disorder and psychiatric stability at the time of the decision.

The notion that social influences, including stigma, shape the shadow of the future through uncertainty is not limited to women with psychiatric disorders. A study on disabled women’s childbirth experiences revealed diverse reactions from their surroundings, leading to heightened fears and a sense of diminished control over their childbirth experiences [42]. Despite the difference in study populations, similar findings indicate a convergence in the experiences of women. While uncertainty about the future during the COVID-19 pandemic has been noted among the general population [28, 29], we specifically examined uncertainty regarding stigma surrounding psychiatric disorders and their potential transmission. Participants' narratives may be influenced by maladaptive prospection seen in persons with psychiatric disorders such as depression and anxiety, which distorts future expectations [43]. Additionally, the potential of transmitting psychiatric disorders to their children might have intensified feelings of uncertainty about the future and thus made the decision-making process more challenging. This phenomenon is not novel and has been documented in various other hereditary diseases [44, 45]. The actual extent of inheritance in psychiatric disorders significantly influences this dynamic. For instance, the estimated heritability for psychotic and neurodevelopmental disorders ranges from 74–85%, whereas for mood and anxiety disorders, it ranges between 37–58% [46]. The high heritability rate of these disorders aligns with the uncertainty as described in the narratives.

### Strengths and limitations

This study provides a nuanced exploration of family planning decision-making in 17 women with psychiatric disorders. The transdiagnostic approach sheds light on overarching issues that were experienced. The use of focus group discussions captured societal dynamics and fostered an interactive environment for reflective perspectives [47, 48]. Thematic framework analysis offered a structured examination of identified themes [29]. However, limitations include the small group of women with unintended pregnancies (*n* = 3) and the retrospective nature of reflections, potentially introducing recall bias [49, 50]. Moreover, it is important to acknowledge the wide age range of participants, as this may affect the consistency of the data. We addressed the potential recall bias by incorporating the reflective nature of our framework and acknowledging its influence on the findings. The iterative process did not include respondent validation of the findings. To mitigate potential misinterpretations, we involved a researcher with lived experience with perinatal mental health problems.

Furthermore, the utilization of focus groups may restrict the depth of individual analyses. Although all our participants had been known with a psychiatric disorder according to the Diagnostic and Statistical Manual of Mental Disorders (DSM-5), we hypothesize that within the diverse array of disorders represented, each psychiatric disorder may have impacted family planning decisions in distinct ways. Additionally, the focus groups did not allow for an in-depth examination of the individual socioeconomic status of the women and how this influenced their reproductive desires. Given the established importance of socioeconomic status in the context of unintended pregnancies [14], it is crucial to consider this factor in individual sessions. Opting for individual interviews could provide a more comprehensive exploration.

### Suggestions for future research

Future research could benefit from longitudinal and prospective study designs, allowing examination of family planning decision-making in women with psychiatric disorders considering the fluctuating aspect of family planning. Distinguishing between various psychiatric disorders and their unique impact on decision-making could provide a more nuanced understanding, possibly through individual in-depth interviews. As women with unintended pregnancies and psychiatric disorders may experience more challenges with family planning decision-making, it would be interesting to include these women in future research.

## Conclusions

Our study sheds light on family planning decisions among women with psychiatric disorders. Like women without psychiatric disorders, past experiences, socioeconomic status, and perceptions on the desire for children shape decision-making. We found that traumatic events have a lasting impact on family planning choices. Stigma, uncertainty about parenting skills, and concerns about transmitting psychiatric disorder(s) contribute to ambivalence about having children. Feelings of regret, grief and relief regarding these decisions reflect the influence of psychiatric disorders. Our results emphasize that women with psychiatric disorders deserve support tailored to their needs, e.g. the possibility to discuss family planning at perinatal mental health facilities. Moreover, healthcare professionals could consider offering ongoing emotional support beyond the reproductive phase to those reflecting on their family planning decisions.

### Supplementary Information

Additional file 1: This file provides the interview guides for the three focus group discussions.

Additional file 2: This file provides the original quotations for each theme per category in Dutch language.

## Data Availability

The dataset(s) supporting the conclusions of this article are included within the article and the additional files. The original data are available upon reasonable request.
