# Supplementary material for: Family planning decision-making in relation to psychiatric disorders in women: a qualitative focus group study
Source: Reprod Health. 2024 Jul 2;21:96. doi: 10.1186/s12978-024-01836-8 (PMC11221133; doi:10.1186/s12978-024-01836-8)
Supplement: Supplementary file 1 — Additional file 1: This file provides the interview guides for the three focus group discussions. [file 12978_2024_1836_MOESM1_ESM.docx]

**Additional file 1: Interview guides**

This file provides the interview guides for the three focus group discussions.

|  | **Focus group 1: Women with unintended pregnancies.** | **Focus group 2: Women without children.** | **Focus group 3: Women with intended pregnancies.** |
| --- | --- | --- | --- |
| **Introduction** | Can you introduce yourself? Why are you participating in this focus group? | Can you introduce yourself? Why are you participating in this focus group? | Can you introduce yourself? Why are you participating in this focus group? |
| **Relation between symptoms and the topic** | (How) have your symptoms contributed to an unintended pregnancy? (planning, physical aspects, sexual aspects) | (How) have your symptoms contributed to the fact that you did not have children?  Was this decision your own? | (How) have your symptoms contributed to an intended pregnancy? (planning, physical aspects, sexual aspects) |
| **Challenges** | Which factors have made the process of fertility, childlessness, and family planning challenging? | Which factors have made the process of fertility, childlessness, and family planning challenging? | Which factors have made the process of fertility, childlessness, and family planning challenging? |
| **Support** | Did you wish for support in this, how, and from whom? | Did you wish for support in this, how, and from whom? | Did you wish for support in this, how, and from whom? |
| **Group specific questions** | Which coping mechanisms have/had helped you (especially during pregnancy)? Do you have suggestions how to turn an unintended pregnancy into an intended pregnancy? | Do you experience societal stigma regarding childlessness and mental health issues? If yes, how do you deal with those? | Which coping mechanisms have/had helped you (during pregnancy and after your baby was born)? |
| **Needs** | What support do women with an unintended pregnancy need? What is the role of the psychiatrist, nurse, general practitioner, or environment? | What support do women need in the decision to not have children? What is the role of the psychiatrist, nurse, general practitioner, or environment? | How can women with mental health issues and a desire to have been supported?  What is the role of the psychiatrist, nurse, general practitioner, or environment? |
| **Control** | Do you experience that you have control over your desire for children and desire for a pregnancy? | Do you experience that you have control over your desire for children and desire for a pregnancy? | Do you experience that you have control over your desire for children and desire for a pregnancy? |
| **Conversation with mental health professionals** | Do you have a recommendation for healthcare providers on how to (optimally) conduct the conversation about family planning? | Do you have a recommendation for healthcare providers on how to (optimally) conduct the conversation about family planning? | Do you have a recommendation for healthcare providers on how to (optimally) conduct the conversation about family planning? |
