# Supplementary material for: Family planning decision-making in relation to psychiatric disorders in women: a qualitative focus group study
Source: Reprod Health. 2024 Jul 2;21:96. doi: 10.1186/s12978-024-01836-8 (PMC11221133; doi:10.1186/s12978-024-01836-8)
Supplement: Supplementary file 2 — Additional file 2: This file provides the original quotations for each theme per category in Dutch language. [file 12978_2024_1836_MOESM2_ESM.docx]

**Additional file 2: Dutch quotations**

This file provides the original quotations for each theme per category in Dutch language.

**Reflections on the decision**

Regret, grief and relief on the decision

57 years, two daughters: “Als ik het heb over mijn psychische kwetsbaarheid, die heb ik jarenlang weggestopt. En vanaf mijn achtenveertigste ben ik eigenlijk bewust van dat ik vanaf mijn jeugd al psychisch kwetsbaar ben. En heeft het een hele kleur over de zwangerschappen en de bevallingen gelegd, ja.”

61 years, one daughter: “Want ik 2012 kreeg mijn dochter, die nu 30 is een ernstige depressie en daar is ze nog steeds niet uit. En als ik dat van tevoren had geweten, ik heb ook depressie, zit ook nu in een depressie. Dan had ik, dan was zij nooit geboren geweest… Als ik had geweten dat ze zo’n ernstige depressie zou krijgen (dat je dat hebt doorgegeven). Ja ik heb dat waarschijnlijk doorgegeven. En dat vind ik heel verschrikkelijk.”

53 years, no children: “Ja en toen wist ik daarna, op een gegeven moment kreeg ik in de gaten dat ik Tourette had, en daar hangt weer, daar hangen van allerlei klachten onder. En toen was ik wel heel erg blij in verband met de erfelijkheid dat ik het niet gedaan heb. Hè het blijft soms toch ook weer, ja het blijft altijd ergens nog een zere plek (ja ja ja). (Heel duidelijk) Ja, heel blij, en heel erg, ehh ja het blijft gewoon jammer soms. (Ja ja)”

70 years, two sons, experienced an unintended pregnancy: “…Als ik nu op mijn leven, ik ben nu zeventig, als ik nu op mijn leven terugkijk, dan waren dat mijn gouden jaren (ja). Het was hartstikke druk en ik moest heel erg zorgen, vier handen tegelijk hebben maar ik heb het wel gedaan.”

59 years, no children: “Doe mij dan maar die doos tissues (huilt) (gelach). Mijn psychiater zegt altijd tegen me, want we hebben het er dus ook over gehad, ja maar je moet het zo zien, je hebt heel goed voor je kinderen gezorgd (hmm, ja). Je hebt, ze goed bewaard. En ze, ja.. (ja, ja)”

**Shadow of the past**

Childhood trauma & adverse life events

62 years, no children, experienced an unintended pregnancy: “Ook al, ook al, het was meer voor mij, ik heb me eigenlijk nooit thuis gevoeld in mijn ouderlijke huis, en ik wilde toch iets van gezin en (…) (ja, hmhm).”

53 years, no children: “En ook van al die diagnoses dacht ik nou, ja, ik geloof toch wel, en ik kom ook uit een

familie, nou niet NSB maar een Duitse een ehh een Duitse familie, gaf ook oorlogstrauma. En dan denk ik van ja, wat ben je allemaal aan het doorgeven (ja), nog los van het technische verhaal van het doorgeven. Dan is dat mijn verantwoording.”

Inadequate parenting

32 years, one son: “ Ja dat had ik ook wel. Dat je wel een voorbeeld had. Ik had wel zoiets van ik wil het niet zo doen als mijn ouders. … Ja je wil het toch anders doen.”

59 years, no children: “… Mijn vader hij had een stemmingsstoornis, manisch depressief. Uhm mijn broer heeft dat ook (hmm). Dat zie ik nu in hem terug. Mijn moeder heeft ADD, of ADHD (ja), dus ik zie het van van twee kanten ehh (ja). Ik zie mijn moeder hysterisch heen en weer rennen. En ik weet dat mijn vader enorme diepe dalen had (ja). En dan zie ik ben achteraf blij dat ik toch ehh (ja) geen ehh… kinderen heb gekregen. (Ja, ja)”

**Shadow of the present**

Awareness of psychiatric disorder(s)

70 years, two sons, experienced an unintended pregnancy: “Eeuhm, ik heb kinderen gekregen op een moment dat ik me nog niet zo heel erg bewust was van mijn psychische aandoeningen. Eeuhm, maar ik wist altijd wel dat ik anders was.”

47 years, two sons: “… toen heb ik mezelf laten diagnosticeren (met autisme), toen werd nog duidelijker, en dat verklaarde ook meteen een hele boel ehh, … daarom dat het ook altijd zo moeizaam ging, ook het moederschap wat mij toch wat lastiger af ging, dan het gros van de ouders van vriendjes.”

Emotions toward psychiatric stability

61 years, one daughter: “Ja ehh… Ik kreeg op mijn achttiende een psychose en toch durfde ik het aan om rond mijn dertigste zwanger te raken. Ik dacht, ik kan dit wel bolwerken.”

40 years, no children: “Uhm, en ik heb altijd geweten voor mij geen kinderen want dan herhaalt de hele bende (recidiverende depressies) zich, ehh dat ga ik niet doen.”

Perceptions on desire for children

70 years, two sons, experienced an unintended pregnancy: “Nee dat was wel, in mijn geval, een bewuste keuze he. Dat mijn eerste zoon kwam. Dus dat uhm… (M: Maar twijfels over de eigen keuze?) De twijfel was een beetje, ja ik wist eigenlijk niet wat ik wilde haha. Ik wist überhaupt niet wat ik met mijn leven wilde.”

62 years, experienced an unintended pregnancy: “Misschien dat ik er daarom emotioneel niet bij kom (unintended pregnancy). Het is wel heel veel (ja). Gewoon getraumatiseerd.”

Status

40 years, no children: “En uhm, maar nu heb ik sinds een jaar of vier een hele stabiele partner (hmm), en nu zit ik toch ook weleens af en toe een beetje te denken, maar ja ik ben veertig dus dus”

**Shadow of the future**

Social influences

24 years, no children: “Ja ook mijn familie, die eh, ik heb ook niet zo veel steun vanuit de familie dus (ja). Dus dan denk ik ja, ja, waarom.”

53 years, no children: “Ja, mijn beste vriendin ook (enorm lastig vinden) die heeft mijn favoriete naam voor een dochter overgenomen (zo), dat is nu twee keer gebeurd. Ja jij krijgt toch geen kinderen. Ja weet je, ik vind dat gewoon zo onattent (ja). En ik ben blij dat ik nu pas weet dat ik autisme heb, ehm, omdat mensen dan iets hebben van, ehm, daar heb je ook al het vooroordeel voor, van ik zou dat niet kunnen (hmm). Terwijl, en dat heeft er eigenlijk niet zoveel mee te maken (hmm). Ik ken genoeg mensen met autisme die uitstekend voor hun kinderen kunnen zorgen.”

age unknown, one son: “En wat ik heel, heel, het was best wel lastig dat, maar ook weer niet heel erg, maar wel een beetje, dat, dat mensen automatisch zeiden van, ervan uitgingen dat je het kind niet wilde houden. Dat vond ik wel lastig. Terwijl ik zoiets had, voor mij is dat helemaal geen vraag. En dan was het van of het allemaal wel verantwoord was terwijl ik zoiets had, dat was voor mij helemaal geen punt.”

Transmission of psychiatric disorder(s)

29 years, no children: “Ja ik zou niet zo’n kind op de wereld willen zetten wat mogelijk een beetje van mij qua psychische klachten meekrijgt.”

53 years, no children: “het is wel een lijn die gestopt moest worden (M: Ja, het verleden, of dat doorspeelt, en of je dan toch iets nieuws voor jezelf wil creëren, toch?)…. Ja daar heb ik nu wel meer zelfvertrouwen en kennis over., dat dat gewoon kan.”

Prospect of motherhood

59 years, no children: “Maar ik denk niet dat ik ze ehm … Ja, goed had kunnen grootbrengen zal ik maar zeggen (ja), in de jaren daarvoor.”

29 years, no children: “en ook ja, kan ik een goede ouder zijn?. Dat vraag ik me ook af, zou ik veel liefde kunnen geven. … Ja, ik denk niet ehh, dat kinderen echt een goede moeder zou kunnen zijn. Dus ja ook een beetje uit bescherming denk ik.”

53 years, no children: “Dat je daar een besluit in kan nemen en dat je met hulp, en… Bij niemand gaat het honderd procent goed. Om mensen zonder enige voorgeschiedenis, ehm, die ehh, ja waar het vreselijk mis gaat. En je weet natuurlijk nooit wat er op je pad komt (ja).”

32 years, one son: “Door de kennis die ik nu heb kan ik wel hard zeggen, ik ben zwaar genetisch belast (ja). En dat is natuurlijk ook iets waar ik me nu meer bewust van ben waar inderdaad ook wel door mijn hoofd gaat van wat als mijn kindje dat krijgt. Aan de andere kant kan ik daar dan wel weer tegenover zetten van ik heb het zelf ook en ik weet nu heel goed hoe ik ermee om kan gaan dus ik herken het waarschijnlijk eerder (ja, ja ja) en ik kan er ook beter in ondersteunen als het zo is (ja). Dat maakt dan ook wel weer dat ik denk ja… (ja). Dat ik dan niet twijfel aan mezelf als moeder ofzo.”
